# Correction: De La Franier et al. Long-Term Reduction of Bacterial Adhesion on Polyurethane by an Ultra-Thin Surface Modifier. *Biomedicines* 2022, *10*, 979

**DOI:** 10.3390/biomedicines12071451

**Published:** 2024-06-28

**Authors:** Brian De La Franier, Dalal Asker, Benjamin Hatton, Michael Thompson

**Affiliations:** 1 Department of Chemistry, University of Toronto, 80 St. George Street, Toronto, ON M5S 3H6, Canada; brian.delafranier@mail.utoronto.ca; 2 Department of Materials Science, University of Toronto, 184 College Street, Toronto, ON M5S 3E4, Canadabenjamin.hatton@utoronto.ca (B.H.); 3 Food Science and Technology Department, Faculty of Agriculture, Alexandria University, Alexandria 21545, Egypt

In the published publication [1], there was an error regarding the affiliations for Dalal Asker. In addition to the following affiliations: (2) Department of Materials Science, University of Toronto; the updated affiliation should include (3) Food Science and Technology Department, Faculty of Agriculture, Alexandria University. Moreover, a permanent email address, dasker10@gmail.com, has been added instead of the single email address listed in the original publication.

There was also an error regarding the affiliation (1) for Brian De La Franier and Michael Thompson. The address “80 St. George Street” was misspelled and now has been corrected.

The authors state that the scientific conclusions are unaffected. This correction has been approved by the Academic Editor, and the original publication has also been updated.
